# Diet and endometrial cancer: a focus on the role of fruit and vegetable intake, Mediterranean diet and dietary inflammatory index in the endometrial cancer risk

**DOI:** 10.1186/s12885-017-3754-y

**Published:** 2017-11-13

**Authors:** Fulvio Ricceri, Maria Teresa Giraudo, Francesca Fasanelli, Dario Milanese, Veronica Sciannameo, Laura Fiorini, Carlotta Sacerdote

**Affiliations:** 1 Department of Clinical and Biological Sciences, University of Turin, Regione Gonzole, 10 Orbassano(TO), Italy; 2 Unit of Epidemiology, Regional Health Service ASL TO3, Via Sabaudia, 164 Grugliasco(TO), Italy; 3 Department of Mathematics “Giuseppe Peano”, University of Turin, Via Carlo Alberto, 10 Turin, Italy; 4 Unit of Cancer Epidemiology, Città della Salute e della Scienza University-Hospital and University of Turin, Via Santena 7, Turin, Italy

**Keywords:** Endometrial cancer, Fruits and vegetables, Mediterranean diet, Dietary inflammatory index, Case-control study

## Abstract

**Background:**

Endometrial cancer is the fourth most common cancer in European women. The major risk factors for endometrial cancer are related to the exposure of endometrium to estrogens not opposed to progestogens, that can lead to a chronic endometrial inflammation. Diet may play a role in cancer risk by modulating chronic inflammation.

**Methods:**

In the framework of a case-control study, we recruited 297 women with newly diagnosed endometrial cancer and 307 controls from Northern Italy. Using logistic regression, we investigated the role of fruit and vegetable intake, adherence to the Mediterranean diet (MD), and the dietary inflammatory index (DII) in endometrial cancer risk.

**Results:**

Women in the highest quintile of vegetable intake had a statistically significantly lower endometrial cancer risk (adjusted OR 5th quintile vs 1st quintile: 0.34, 95% CI 0.17-0.68). Women with high adherence to the MD had a risk of endometrial cancer that was about half that of women with low adherence to the MD (adjusted OR: 0.51, 95% CI 0.39-0.86). A protective effect was detected for all the lower quintiles of DII, with the highest protective effect seen for the lowest quintile (adjusted OR 5th quintile vs 1st quintile: 3.28, 95% CI 1.30-8.26).

**Conclusions:**

These results suggest that high vegetable intake, adherence to the MD, and a low DII are related to a lower endometrial cancer risk, with several putative connected biological mechanisms that strengthen the biological plausibility of this association.

**Electronic supplementary material:**

The online version of this article (10.1186/s12885-017-3754-y) contains supplementary material, which is available to authorized users.

## Introduction

Endometrial cancer is the fourth most common cancer in European women, [1] with about 56,000 new cases diagnosed in 2008. [2] The major risk factor for endometrial cancer is an unbalanced and/or prolonged exposure of the endometrium to oestrogens. Indeed, exposure to endogenous or exogenous oestrogens not opposed by progestogens leads to an increase in the mitotic activity of endometrial cells, resulting in an increase in DNA replication and an increased probability of somatic mutations [3]. Such unbalanced or prolonged exposure can occur in women who experience late menopause, are nulliparous, have polycystic ovary syndrome, take oestrogen replacement therapy (without progestogens), or are overweight/obese [3].

Furthermore, the hormonal regulation of the growth and shedding of the endometrial mucosa during the menstrual cycle is associated with endometrial inflammation, [4]. which can be aggravated by hormonal deregulation. Chronic endometrial inflammation is also associated with overweight and obesity. This raises the possibility that local inflammation may be a risk factor in endometrial cancer development, [5] and by extension, that diet may play a role independently of obesity by mediating oestrogen levels [3] and/or by modulating chronic inflammation. The evidence of an association between endometrial cancer risk and specific dietary components is limited and includes a publication from the World Cancer Research Fund, which reported a probable beneficial association between coffee consumption and endometrial cancer risk, as well as a possible negative association with glycaemic load [6]. In this conceptual framework, we examined the role of fruit and vegetable intake, adherence to the Mediterranean diet (MD), and the dietary inflammatory index (DII) in endometrial cancer risk.

## Methods

### Subjects

Endometrial cancer cases were recruited at the Turin University Gynaecological Hospital, where about 70% of endometrial cancers occurring in the Piedmont Region of Northwest Italy area are treated. We recruited 297 women who lived in the Piedmont Region (age 40–74 years) and were newly diagnosed with histologically confirmed endometrial cancer.

Two sets of controls were recruited: i) a random sample of females (age 40–74 years) from the Turin centre of the European Prospective Investigation into Cancer and Nutrition (EPIC-Turin population controls, *N* = 98) [7]; and ii) a hospital-based sample of women (age 40–74 years) treated at the general university hospital for minor afflictions not related to diet or to hormonal status (*N* = 209). Both control groups were made up of women residing the Piedmont Region who had not undergone hysterectomy. The two control groups were comparable with respect to the distribution of major confounding factors (data not shown).

The research have been approved by the Human Genetics Foundation (HuGeF) and University of Turin ethical committee and all subjects enrolled in the study signed an informed consent form.

### Data collection

The information analyzed in this study was collected using questionnaires from EPIC Italy: a lifestyle questionnaire and a validated food frequency questionnaire (FFQ). Age, age at menarche, parity, oral contraceptive use, menopausal status, use of hormone replacement therapy, body mass index (BMI), physical activity (occupational and recreational), education, tobacco smoking status, diet and education was taken from the EPIC Italy lifestyle questionnaire [7]. Women were classified as postmenopausal if they had gone at least 1 year without any menstrual cycle. Physical activity was categorised as inactive or moderately inactive (<10 h/week), moderately active (10–24 h/week), and active (>24 h/week). An additional, short questionnaire was used to collect more detailed information on hormonal and reproductive history and physical activity. The estimated intake and average portion sizes of up to 260 food items consumed in the last 12 months was taken from the validated EPIC Italy FFQ [8]. A matrix for the conversion of food items into nutrients and micronutrients was applied [9].

A trained interviewer administered the questionnaires to cases and hospital-based controls during a face-to-face interview. During the questionnaire interviews, measures of weight, height, and waist and hip circumferences were taken for all subjects, and before the beginning of any cancer treatment among cases.

Information on the EPIC-Turin population controls was taken from the same EPIC Italy questionnaires. These questionnaires were administered in an identical manner, and the same anthropometric measures were taken, but these steps were completed at the time of enrolment in the EPIC Study.

### Dietary indices

The dietary habits of the women included in the study were summarised in two indices: an MD index and a DII index. The MD index was constructed based on women’s adherence to MD as per Trichopoulou et al., [10] using food groups recommended by Davidson and Passmore [11]. The MD index takes into account eight dietary habits common to the MD: high monounsaturated/saturated fat consumption ratio, high consumption of legumes, high consumption of cereals (including bread and potatoes), high consumption of fruits, high consumption of vegetables, moderate ethanol consumption (less than two glass of wine a day, but not abstainer), low consumption of meat and meat products, and low consumption of milk and dairy products. Median values were used as the cut-off (Table 1 Panel A). Women were divided into three categories according to the number of habits adopted: low adherence to the MD (from 0 to 3 habits), moderate adherence to the MD (from 4 to 5 habits), or high adherence to the MD (more than 6 habits).

**Table 1:** Data used to build dietary indices. Panel A: Median (IQR) of food or nutrient intakes used as cut-off for the Mediterranean diet index. Panel B: Mean (SD) of food or nutrient intakes and overall inflammatory effect score used for the dietary inflammation index

Panel A. Mediterranean diet index		
Food group or nutrient (g/day)	Median (IQR)	Score
Legumes	81.40 (54.80–127.20)	+1 above the median
Cereals	74.70 (40.90–133.90)	+1 above the median
Fruits	253.20 (184.60–345.60)	+1 above the median
Vegetables	81.40 (54.80–127.20)	+1 above the median
Meat and meat products	100.20 (68.70–134.30)	+1 below the median
Milk and dairy products	148.70 (58.30–227.10)	+1 below the median
Monounsaturated/saturated fat ratio	1.45 (1.25–1.69)	+1 above the median
Ethanol consumption	9.60 (0.00–125.00)	+ 1 not abstainer and less than 24 g/day
Panel B, dietary inflammation index		
Food group or nutrient	Mean (SD)	Overall inflammatory effect score
β-Carotene (μg)	3166.13 (1783.27)	−0.584
Caffeine (g)	35.91 (23.24)	−0.124
Carbohydrate (g)	218.48 (80.03)	0.109
Cholesterol (mg)	311.08 (129.26)	0.347
Energy intake(Kcal)	1825.98 (565.17)	0.180
Total fat (g)	73.35 (25.33)	0.298
Fibre (g)	18.80 (6.19)	−0.663
Folic Acid (μg)	253.26 (88.03)	−0.207
Ferrum (mg)	11.67 (3.49)	0.032
Garlic (g)	2.90 (2.95)	−0.412
MUFA (g)	35.70 (12.66)	−0.019
Niacin(mg)	15.57 (4.68)	−1.00
*N*-3 Fatty acid (g)	1.08 (0.38)	−0.436
*n-*6 Fatty acid (g)	7.10 (3.28)	−0.159
Onion	8.71 (7.91)	−0.301
Protein(g)	75.44 (23.16)	0.021
PUFA (g)	8.76 (3.72)	−0.337
Riboflavin (mg)	1.39 (0.48)	−0.727
Saturated fat (g)	24.74 (9.95)	0.429
Tea (g)	45.93 (81.72)	−0.536
Thiamin (mg)	0.87 (0.27)	−0.354
Vitamin A (RE)	1047.19 (683.17)	−0.401
Vitamin B6 (mg)	1.64 (0.50)	−0.365
Vitamin C (mg)	134.39 (68.63)	−0.424
Vitamin D (μg)	2.38 (1.22)	−0.446
Vitamin E (mg)	7.41 (2.93)	−0.419
Zinc (mg)	10.37 (3.43)	−0.313

*IQR* interquartile ratio, *SD* standard deviation

A DII was then derived based on the original DII by Cavicchia et al [12] and its successive improvement by Shivappa et al [13]. We evaluated the consumption of the available items from the FFQ: twenty-four nutrients (β-carotene, caffeine, carbohydrates, cholesterol, total energy intake, total fat, fibre, folic acid, ferrum, MUFA, niacin, *N*-3 fatty acid, *n-*6 fatty acid, protein, PUFA, riboflavin, saturated fat, thiamin, vitamin A, vitamin B6, vitamin C, vitamin D, vitamin E, and zinc), and three available food items (garlic, onion, and tea), and we weighted their intake using the overall inflammatory effect scores as computed by Shivappa et al [13] (Table 1 Panel B).

### Statistical analyses

Preliminary data analysis was performed using the mean and standard deviation (SD) or the frequency and percentage for quantitative or qualitative variables, respectively. The intake of fruits and vegetables was divided into quintiles of consumption (using the distribution of controls).

We used the Wilcoxon rank sum test with continuity correction and the Chi-squared test to determine differences in general factors and in food and nutrient groups between cases and controls. Odds ratios (OR) and corresponding 95% confidence intervals (CI) were computed using unconditional logistic regression models, both univariate and multivariate, adjusting for age, age at menarche, parity, oral contraceptive use, menopausal status, use of hormone replacement therapy, BMI, physical activity, education, smoking status, and total energy intake.

Subgroup analyses were also carried out among normal weight women (i.e., BMI < 25 kg/m^2^), overweight women (i.e., BMI 25–30 kg/m^2^), and obese women (i.e., BMI >30 kg/m^2^), and sensitivity analyses were performed among the two control groups to exclude discrepancies in the results obtained for these groups. All the analyses were performed using SAS V9.2 package (SAS Inc., Cary, NC, USA).

## Results

Cases (*N* = 297) and controls (*N* = 307) were comparable with respect to age, with a mean age at interview of 61.49 (SD 7.48) years for cases and 60.40 (SD 7.72) years for controls. The group of endometrial cancer cases included more nulliparous women (15.41% vs 5.61%) when compared to controls, as well as fewer patients with a parity ≥2 (6.45% vs 21.05%). Among endometrial cancer cases there was a higher percentage of women with lower education (40.94% with primary school or less vs 27.27% for controls) and a slightly higher percentage of never-smokers (67.99% vs 61.15%). Among controls there was a lower percentage of postmenopausal women (83.89% vs 93.93% for cases), a lower mean age at menarche (12.51 years, SD 1.51 vs 12.75 years, SD 1.53), and a lower mean BMI (26.61 kg/m^2^, SD 16.82 vs 28.01 kg/m^2^, SD 5.90). Parity (*p*-value < 0.0001), menopausal status (*p*-value = 0.0001), BMI (*p* < 0.0001), and education (*p*-value = 0.001) showed the most evident differences (Table 2).

**Table 2:** Distribution of characteristics among endometrial cancer cases and controls (means and standard deviation or frequencies and percentages)

General characteristics	Cases	Controls	*p*-value^a^
	(n = 297)	(n = 307)	
Age (years)	61.49 (7.48)	60.40 (7.72)	0.10
Age at menarche (years)	12.75 (1.53)	12.51 (1.51)	0.53
Parity			<0.001
0	43 (15.41%)	16 (5.61%)	
1	218 (78.14%)	209 (73.33%)	
≥ 2	18 (6.45%)	60 (21.05%)	
Oral contraceptive use			0.39
Yes	53 (19.00%)	65 (21.89%)	
No	226 (81.00%)	232 (78.11%)	
Menopausal status			0.0001
Postmenopausal	263 (93.93%)	250 (83.89%)	
Premenopausal	17 (6.07%)	48 (16.11%)	
Hormone replacement therapy			0.36
Yes	52 (18.64%)	64 (21.62%)	
No	227 (81.36%)	232 (78.38%)	
Body mass index (kg/m^2^)			<0.001
< 25 (normal weight)	107 (36.03%)	163 (53.09%)	
25–30 (overweight)	95 (31.99%)	98 (31.92%)	
> 30 (obese)	95 (31.99%)	46 (14.98%)	
Physical activity			0.08
Inactive or moderately inactive	28 (10.22%)	47 (16.10%)	
Moderately active	161 (58.76%)	169 (57.88%)	
Active	85 (31.02%)	76 (26.03%)	
Education			0.001
Primary school or less	113 (40.94%)	81 (27.27%)	
Secondary or vocational school	106 (38.41%)	125 (42.09%)	
High school or more	57 (20.65%)	91 (30.64%)	
Smoking status			0.04
Never smoker	189 (67.99%)	181 (61.15%)	
Former smoker	68 (24.46%)	73 (24.66%)	
Current smoker	21 (7.55%)	42 (14.19%)	
Alcohol consumption (g/day)	6.91 (9.81)	5.87 (9.95)	0.56
Fruit consumption (g/day)	262.87 (140.98)	289.35 (146.28)	0.03
Vegetable consumption (g/day)	85.24 (50.62)	112.24 (74.49)	<0.0001
Total energy intake (Kcal/day)	7569 (2319)	7706 (2408)	0.60
Mediterranean diet index			0.0003
Low adherence (0–3 habits)	158 (53.20%)	115 (37.46%)	
Moderate adherence (4–5 habits)	111 (37.37%)	143 (46.58%)	
High adherence (6–8 habits)	28 (9.43%)	49 (15.96%)	
Dietary index of inflammation			0.33
1st quintile (low inflammation)	46 (16.49%)	69 (23.31%)	
2nd quintile	56 (20.07%)	59 (19.93%)	
3rd quintile	57 (20.43%)	58 (19.59%)	
4th quintile	60 (21.51%)	55 (18.58%)	
5th quintile (high inflammation)	60 (21.51%)	55 (18.58%)	

^a^Wilcoxon or Chi-squared test

We found a highly significant (*p* < 0.0001) lower vegetable intake among cases (mean 85.24 g/day, SD 50.62) with respect to controls (mean 112.24 g/day, SD 74.49) and a less pronounced, lower fruit intake (mean 262.87 g/day, SD 140.98 vs mean 289.35 g/day, SD 146.28), while no association was found with alcohol intake or total energy intake (Table 2).

A significant protective effect was found for high vegetable intake (adjusted OR 5th quartile vs 1st quartile: 0.34, 95% CI 0.17–0.68, *p*-value for trend = 0.0003), and a possible protective effect of high fruit intake was suggested (adjusted OR 5th quartile vs 1st quartile: 0.55, 95% CI 0.28–1.06, *p*-value for trend = 0.08). Both moderate (adjusted OR: 0.57, 95% CI 0.39–0.86) and high (adjusted OR: 0.51, 95% 0.28–0.92) adherence to the MD resulted in a reduction in endometrial cancer risk of about 50%. A borderline trend (*p*-value = 0.06) of increasing risk for increasing DII was found, with highly significant results obtained for the 2nd, 3rd, and 4th quintiles with respect to the 1st quintile (Table 3).

**Table 3:** Odds ratios (OR) and 95% confidence Intervals (CI) by fruit and vegetable quintiles, Mediterranean diet index, and dietary inflammation index quintiles

	Crude OR^a^	95% CI	Adjusted OR^b^	95% CI
FRUIT				
1st quintile	Reference	–	Reference	**–**
2nd quintile	0.85	0.52–1.39	0.73	0.41–1.28
3rd quintile	0.71	0.43–1.18	0.60	0.33–1.09
4th quintile	0.77	0.45–1.27	0.66	0.36–1.21
5th quintile	0.54	0.32–0.92	0.55	0.28–1.06
p-value for trend	0.03		0.08	
VEGETABLES				
1st quintile	Reference	–	Reference	**–**
2nd quintile	1.18	0.73–1.89	1.11	0.65–1.92
3rd quintile	0.58	0.35–0.98	0.55	0.31–0.98
4th quintile	0.65	0.39–1.08	0.58	0.31–1.05
5th quintile	0.29	0.16–0.52	0.34	0.17–0.68
p-value for trend	<0.0001		0.0003	
MEDITERRANEAN DIET INDEX				
Low adherence (0–3 habits)	Reference	–	Reference	**–**
Moderate adherence (4–5 habits)	0.58	0.40–0.82	0.57	0.39–0.86
High adherence (6–8 habits)	0.43	0.25–0.72	0.51	0.28–0.92
p-value for trend	0.0002		0.004	
DIETARY INDEX OF INFLAMMATION				
1st quintile	Reference	–	Reference	**–**
2nd quintile	1.62	0.94–2.80	2.77	1.41–5.44
3rd quintile	1.51	0.85–2.62	2.44	1.17–5.09
4th quintile	1.73	1.01–2.97	3.03	1.35–6.76
5th quintile	1.79	1.04–3.07	3.28	1.30–8.26
p-value for trend	0.06		0.06	

^a^univariate analysis

^b^multivariate logistic regression models adjusted for age, parity, menopausal status, hormone replacement therapy use, oral contraceptive use, body mass index, age at menarche, physical activity, education, smoking status, and total energy intake

Both the subgroup analyses in normal weight, overweight, and obese women (Additional file 1: Table S1) and the sensitivity analyses among the two control groups (data not shown) showed the same trends we observed in our principal analyses, with a lower statistical significance due to the reduction in sample size.

## Discussion

In the present study, we analysed the possible role of diet in the incidence of endometrial cancer. In particular, we explored the role of dietary patterns that may mediate oestrogen levels and modulate chronic inflammation. High vegetable intake, high adherence to the MD and DII showed a protective effect on endometrial cancer risk.

These results are in agreement with previous studies [14, 15] and show a clear protective effect of vegetable intake on endometrial cancer risk and a less compelling protective effect of fruit intake. Vegetables, and in particular non-starchy vegetables, may protect from cancer through modulation of steroid hormone concentrations and metabolism, activation of antioxidant mechanisms, modulation of detoxification enzymes, and stimulation of the immune system [16, 17].

We used two validated dietary indices under the hypothesis that the use of such indices, which take into account the interactions among various combinations of foods and nutrients, could be a stronger determinant of endometrial cancer risk than any single dietary component. The MD is rich in phytoestrogens, agents with oestrogen-like effects that may compete with oestrogens in binding to oestrogen receptors, thus exerting antioestrogenic effects [18]. Furthermore, the MD contains several antioxidants with important anti-inflammatory properties that have been inversely related with cancer risk in previous case-control studies [19].

Despite mixed results from a recent meta-analysis, which could not demonstrate an association between significantly lower endometrial cancer risks and higher adherence to the MD, [20] our study supports the evidence for a protective effect of the MD on endometrial cancer risk.

A clear protective effect of a lower DII was observed in this study. Previous studies have indicated that foods such as coffee and vegetables are inversely related to endometrial cancer risk, and are thus consistent with the hypothesis that an antioxidant diet could be positively involved in cancerogenesis. All these dietary factors contribute to lower DII values, while animal products, saturated fat acids, and starches contribute to higher DII values. A recent, large case-control study showed a positive association between DII and endometrial cancer [20]. In particular, the authors found that the OR for women in the highest quartile of DII versus women in the lowest quartile was 1.46 (95% CI 1.02–2.11) with a *p*-value for trend of 0.04.

Inflammation has been related to endometrial cancer both in cohort and case-control studies. In the EPIC study and in the Women’s Health Initiative, C-reactive protein (CRP) and other pro-inflammatory cytokines (such as IL1 receptor antagonist) were found to be positively associated with endometrial cancer [21–23].

Consumption of pro-inflammatory foods (such as animal products) seems to increase CRP levels [24]. This can cause chronic subclinical inflammation which may lead to an increase in insulin resistance, [25] which in turn could be responsible for the stimulation of cell proliferation and the inhibition of apoptosis [3].

The present study has some limitations that are inherent to the case-control design. Potential selection and information bias should be considered. The participation rate for both cases and controls was higher than 95%, and we excluded all women with diagnoses that could be related to known risk factors for endometrial cancer from the control group, as well as patients with previous hysterectomy. Recall bias was possible due to the case-control design; however the hypothesis of a dietary aetiology for endometrial cancer is not well known in the general population. The comparability of recall between cases and the hospital-based controls was improved by interviewing them in a hospital setting. In spite of these limitations, the study has some strengths, such as accurate exposure assessment with a validated questionnaire, and the possibility to adjust the analysis for several known confounders.

In conclusion, the present case-control study provided some evidence that high vegetable intake, adherence to the MD, and a low DII are related to a lower endometrial cancer risk, with several putative connected biological mechanisms that strengthen the biological plausibility of this association.

## Additional file

Additional file 1: Table S1. Odds ratios (OR) and 95% confidence Intervals (CI) by fruit and vegetable quintiles, Mediterranean diet index, and dietary inflammation index quintiles. (PDF 8 kb)
