# Supplementary material for: Diet and endometrial cancer: a focus on the role of fruit and vegetable intake, Mediterranean diet and dietary inflammatory index in the endometrial cancer risk
Source: BMC Cancer. 2017 Nov 13;17:757. doi: 10.1186/s12885-017-3754-y (PMC5683600; doi:10.1186/s12885-017-3754-y)
Supplement: Additional file 1: Table S1. — Odds ratios (OR) and 95% confidence Intervals (CI) by fruit and vegetable quintiles, Mediterranean diet index, and dietary inflammation index quintiles. (PDF 8 kb) [file 12885_2017_3754_MOESM1_ESM.pdf]

Supplementary Table 1. Odds ratios (OR) and 95% confidence Intervals (CI) by fruit and vegetable quintiles, Mediterranean diet index, and dietary inflammation index quintiles.
***univariate analysis**
****multivariate logistic regression models adjusted for age, parity, menopausal status, hormone replacement therapy use, oral contraceptive use, body mass index, age at menarche, physical activity, education, smoking status, and total energy intake.**

Normal weight (BMI <25)					Overweith (BMI 25-30)					Obese (BMI >30)				
	**Crude OR***	**95% CI**	**Adjusted OR****	**95% CI**		**Crude OR***	**95% CI**	**Adjusted OR****	**95% CI**		**Crude OR***	**95% CI**	**Adjusted OR****	**95% CI**
**FRUIT**														
1st quintile	**Reference**	-	**Reference**	-	1st quintile	**Reference**	-	**Reference**	-	1st quintile	**Reference**	-	**Reference**	-
2nd quintile	1.20	0.56-2.61	0.89	0.38-2.07	2nd quintile	0.81	0.34-1.98	1.03	0.36-2.98	2nd quintile	0.46	0.16-1.33	0.33	0.08-1.33
3rd quintile	0.94	0.43-2.05	0.74	0.31-1.79	3rd quintile	0.81	0.33-2.00	0.62	0.22-1.78	3rd quintile	0.39	0.12-1.19	0.38	0.08-1.73
4th quintile	0.72	0.32-1.59	0.41	0.16-1.06	4th quintile	0.94	0.40-2.23	1.40	0.48-4.06	4th quintile	0.88	0.25-3.15	0.43	0.09-2.07
5th quintile	1.00	0.46-2.20	0.72	0.28-1.85	5th quintile	0.37	0.14-0.99	0.70	0.20-2.42	5th quintile	0.35	0.10-1.16	0.29	0.06-1.45
p-value for trend	0.58		0.19		p-value for trend	0.13		0.85		p-value for trend	0.23		0.24	
**VEGETABLES**														
1st quintile	**Reference**	-	**Reference**	-	1st quintile	**Reference**	-	**Reference**	-	1st quintile	**Reference**	-	**Reference**	-
2nd quintile	0.87	0.42-1.77	0.77	0.34-1.77	2nd quintile	2.47	1.04-5.84	3.12	1.16-8.38	2nd quintile	1.04	0.29-3.69	0.73	0.18-2.96
3rd quintile	0.55	0.25-1.24	0.41	0.17-1.02	3rd quintile	1.02	0.40-2.58	1.71	0.59-4.99	3rd quintile	0.28	0.09-0.86	0.31	0.08-1.26
4th quintile	0.75	0.35-1.61	0.54	0.21-1.38	4th quintile	1.08	0.43-2.72	1.40	0.48-4.10	4th quintile	0.27	0.08-0.84	0.27	0.06-1.24
5th quintile	0.29	0.12-0.70	0.32	0.11-0.92	5th quintile	0.42	0.14-1.31	0.49	0.13-1.81	5th quintile	0.20	0.05-0.73	0.26	0.04-1.56
p-value for trend	0.01		0.02		p-value for trend	0.03		0.16		p-value for trend	0.001		0.06	
**MEDITERRANEAN DIET INDEX**														
Low adherence (0-3 habits)	**Reference**	-	**Reference**	-	Low adherence (0-3 habits)	**Reference**	-	**Reference**	-	Low adherence (0-3 habits)	**Reference**	-	**Reference**	-
Moderate adherence (4-5 habits)	0.74	0.43-1.28	0.75	0.41-1.39	Moderate adherence (4-5 habits)	0.48	0.26-0.90	0.54	0.34-2.53	Moderate adherence (4-5 habits)	0.47	0.21-1.04	0.37	0.14-0.99
High adherence (6-8 habits)	0.38	0.16-0.89	0.33	0.13-0.84	High adherence (6-8 habits)	0.65	0.27-1.54	0.93	0.34-2.53	High adherence (6-8 habits)	0.31	0.09-1.09	0.37	0.07-2.07
p-value for trend	0.03		0.03		p-value for trend	0.11		0.45		p-value for trend	0.03		0.06	
**DIETARY INDEX OF INFLAMMATION**														
1st quintile	**Reference**	-	**Reference**	-	1st quintile	**Reference**	-	**Reference**	-	1st quintile	**Reference**	-	**Reference**	-
2nd quintile	1.19	0.51-2.74	2.35	0.82-6.73	2nd quintile	2.14	0.81-5.63	2.55	0.78-8.37	2nd quintile	2.06	0.65-6.51	1.97	0.45-8.69
3rd quintile	0.70	0.30-1.64	1.11	0.34-3.60	3rd quintile	2.97	1.10-7.99	4.37	1.18-16.2	3rd quintile	3.71	1.02-13.5	2.37	0.46-12.2
4th quintile	1.01	0.42-2.41	1.74	0.48-6.36	4th quintile	2.40	0.96-6.01	3.77	0.94-15.2	4th quintile	3.12	0.95-10.2	2.96	0.47-18.5
5th quintile	0.88	0.38-2.01	1.56	0.37-6.54	5th quintile	3.65	1.32-10.1	4.46	0.82-24.3	5th quintile	2.62	0.87-7.88	1.72	0.21-14.3
p-value for trend	0.63		0.86		p-value for trend	0.02		0.11		p-value for trend	0.08		0.55	
